# Conditions promoting effective very high gravity sugarcane juice fermentation

**DOI:** 10.1186/s13068-018-1239-0

**Published:** 2018-09-18

**Authors:** Bruno Monteiro, Pedro Ferraz, Mário Barroca, Sandra H. da Cruz, Tony Collins, Cândida Lucas

**Affiliations:** 1 Laboratory of Food and Beverage Biotechnology, ESALQ, University of São Paulo, Piracicaba, SP 13418-900 Brazil; 2 Centre of Molecular and Environmental Biology (CBMA), University of Minho, 4710-057 Braga, Portugal; 3 Institute of Science and Innovation for Bio-Sustainability (IB-S)/CBMA, University of Minho, Campus de Gualtar, 4710-057 Braga, Portugal

**Keywords:** Biofuel, Bioethanol, Sugarcane, *Saccharomyces cerevisiae*, CAT-1, Very high gravity, Process optimisation, Process sustainability

## Abstract

**Background:**

Applying very high gravity (VHG) fermentation conditions to the sugarcane juice (SCJ) bioethanol industry would improve its environmental and economic sustainability without the need for major infrastructure changes or investments. It could enable a decrease in the consumption of biological and natural resources (cane/land, water and energy) while maintaining acceptable production parameters. The present study attempts to demonstrate and characterise an effective industrially relevant SCJ-VHG fermentation process.

**Results:**

An industry-like SCJ-VHG bioethanol production process with 30 and 35 °Bx broth was employed to investigate the effects of both the yeast strain used and nitrogen source supplementation on process yield, process productivity, biomass viability, glycerol concentration and retention-associated gene expression. Process performance was shown to be variably affected by the different process conditions investigated. Highest process efficiency, with a 17% (w/v) ethanol yield and only 0.2% (w/v) sugar remaining unfermented, was observed with the *Saccharomyces cerevisiae* industrial strain CAT-1 in 30 °Bx broth with urea supplementation. In addition, efficient retention of glycerol by the yeast strain was identified as a requisite for better fermentation and was consistent with a higher expression of glycerol permease *STL1* and channel *FPS1*. Urea was shown to promote the deregulation of *STL1* expression, overcoming glucose repression. The consistency between Fps1-mediated ethanol secretion and ethanol in the extracellular media reinforces previous suggestions that ethanol might exit the cell through the Fps1 channel.

**Conclusions:**

This work brings solid evidence in favour of the utilisation of VHG conditions in SCJ fermentations, bringing it a step closer to industrial application. SCJ concentrated up to 30 °Bx maintains industrially relevant ethanol production yield and productivity, provided the broth is supplemented with a suitable nitrogen source and an appropriate industrial bioethanol-producing yeast strain is used. In addition, the work contributes to a better understanding of the VHG-SCJ process and the variable effects of process parameters on process efficiency and yeast strain response.
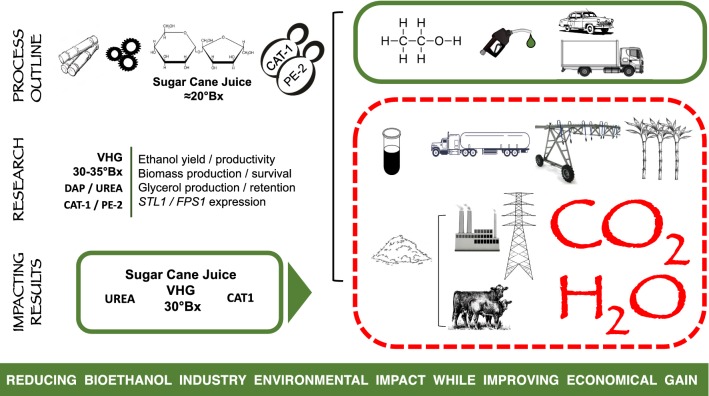

## Background

Sugar crops such as sugarcane, sugar beet and sweet sorghum are the principal feedstocks used in industrial bioethanol production today. They account for almost 60% of global production, with starch crops and principally corn accounting for most of the remainder, and with the relatively recently introduced second and third generation feedstocks (lignocellulosic materials, algae, etc.) currently accounting for only a minor proportion. Brazil is the world’s largest producer of sugar-based bioethanol with ~ 30 billion litres being produced per year (statistics available at SugarCane.org). This is produced from sugarcane juice (SCJ) via yeast fermentation with sugar concentrations of up to ~ 20 °Bx (°Brix = g sugar/100 g solution) and with ~ 10–17% w/v yeast wet biomass, without temperature control. Most typically, the production process involves consecutive 8 h fermentation cycles in which the yeast biomass is recycled between cycles by centrifugation and washing in sulphuric acid (pH 2) to eliminate bacterial contamination [1, 2] before subsequent reuse in inoculation of the next cycle. This cycle lasts uninterrupted for 3–4-months.

The SCJ industrial fermentation process was first implemented in Brazil in the 1980s and has undergone only moderate technological improvements since then [3]. The sucrose–ethanol conversion yield is already relatively high, at around 90–92%, but considering the dimension of the industry, which supports Brazil as one of the largest bioethanol producers in the world, any further yield enhancements or technological or operational advances should allow for significant benefits. Indeed, the current process is characterised by a reduced economic and environmental sustainability due to high energy and fresh water consumption as well as high biowaste output (vinasse and bagasse). Approximately 10–13 L of vinasse are produced per litre of distilled ethanol. This potassium rich biowaste is used in sugar cane field fertilisation [4], but the costs associated with the storage and distribution logistics of this solution and the amounts of vinasse currently generated have reached such high levels as to constitute a waste-disposal problem with high environmental and economic impact [4]. Bagasse, on the other hand, is burnt in situ for heat generation used to feed the SCJ concentrator evaporators as well as thermoelectric plants producing electricity.

Very high gravity (VHG) fermentation technology (reviewed in [5, 6]), which is based on the use of higher concentrations of sugar substrate than presently used, offers the potential of reducing the environmental and economic impact of bioethanol production from SCJ. It is a versatile technology which can be easily applied to existing industrial facilities and enables high savings in process water and energy requirements as well as enhanced process productivity and efficiency. This leads to reduced vinasse production, a more efficient use of fermenter space and an ameliorated process energy balance [6–9]. Indeed, energy savings up to 4% have been reported [3, 6], with the energy needed for concentration of the SCJ to the higher levels required being obtained from the existing bagasse biowaste burning process and evaporation systems [2, 6, 9]. In addition, the high sugar and ethanol levels, and the resultant higher osmotic stress, characteristic of the process, lead to reduced contamination problems and hence the potential of a reduced need for inter-cycle acid washing [6].

Recognizably, one of the biggest challenges posed by the VHG process is related to the harsh process conditions used and the maintenance of yeast viability and performance under these conditions. This has led to the pursuit of yeast strains that tolerate extremely high osmotic stress while also efficiently producing very high amounts of ethanol and utilising all available sugar, in addition to retaining high viability over the whole fermentation period [10, 11]. Currently, the *Saccharomyces cerevisiae* strains PE-2 and CAT-1 are the most commonly used in industrial SCJ fermentations in Brazil. These strains originate from the extreme environments of bioethanol plants [10, 11] where confined evolution and selection has favoured the fixation of phenotypes of high resistance and productivity and thus are better suited to the harsh industrial bioethanol production conditions. Indeed, under the high osmotic stress of standard fermentation conditions (~ 20 °Bx), strain PE-2 was reported to yield 9.1% (w/v) ethanol with 3% (w/v) glycerol remaining and a productivity of 92%, while retaining high (94%) cell viability and high biomass productivity [10, 11].

Typically, yeasts counterbalance osmotic stress by glycerol/osmolyte accumulation [12] via activation of glycerol metabolism and/or glycerol retention [13], these being under the regulation of several intertwining signalling pathways, including HOG, CWI, TOR and Ras (reviewed by [14]). Glycerol retention is achieved through closure of the glycerol plasma membrane channel Fps1 [15–17], and increasing the glycerol influx from the extracellular medium via the glycerol active permease Stl1 [16, 18]. On the other hand, increased glycerol production derives from increased expression of the cytosolic enzyme glycerol-3-phosphate dehydrogenase (Gpd1) which converts dihydroxyacetone phosphate (DHAP) into glycerol 3-phosphate (G3P) [19]. Gpd1 operates the glycerol-3P shuttle in concert with the mitochondrial glycerol-3-phosphate dehydrogenase (Gut2) from the glycerol consumption pathway, coupling the cytosolic NAD(H) and the mitochondrial FAD(H) redox pools [20, 21]. In regular conditions, the G3P produced is therefore re-transformed into DHAP by Gut2, but it is also channelled for lipid synthesis [22]. Under osmotic stress, G3P is additionally substrate of the glycerol phosphatase Gpp2 to produce high amounts of glycerol [23]. While these processes are mandatory for cell survival, they expectably decrease the fermentative flux [19] and hence ethanol yield, especially under osmotic stress [16]. Expectably, good industrial fermentation yeasts should be able to fine tune the balance between these processes, to survive the stress whilst achieving highest bioethanol production yields. Indeed, this is well documented for wine strains in high osmotic stress conditions [24], but not for bioethanol fermentations.

Supplementation of fermentation media with various nutrients, namely with a nitrogen-rich source, has been investigated for enhancing yeast cell performance and survival under high osmotic stress conditions [16]. For example, supplementation with an ammonium source has been shown to overcome reduced ethanol production [25–27] and circumvent slow or stuck wine fermentations [28, 29], while it was reported to increase glycerol production in anaerobic fermentations due to redox balance requirements [24, 30, 31]. In particular, a diammonium phosphate (DAP) concentration equivalent to 300 mg L^−1^ nitrogen was shown to enhance yeast viability and fermentation ability in grape must fermentations at high temperatures [26, 28, 32]. Another nitrogen source, urea, has also been reported to improve yeast performance and survival with various substrates and fermentation conditions [7, 25], including VHG fermentations of molasses [8]. In this latter case, the need for further osmoprotectants (such as soy flour) was occasionally recommended for optimal yeast performance [33]. Importantly, it must be also noted that nitrogen supplementation has also been reported to sometimes lead to detrimental effects such as the triggering of nitrogen catabolite repression (NCR) [26, 34], or the production of ethyl carbamate from ethanol [35, 36] or other undesirable compounds [37]. Moreover, the yeasts themselves can variably affect the available nitrogen concentrations during fermentation [27], as, depending on the process conditions and yeast strain, the nitrogen compounds can be produced and/or assimilated.

VHG technology has already been studied with diverse fermentable substrates at the laboratory scale [7–9, 32], but its use in industrial processes with SCJ and industrial yeast strains is much less investigated. In particular, SCJ has been used mixed with molasses [9]. This nevertheless requires that molasses is taken from its regular use in sugar refining, or imported if the plant does not produce sugar, either of which increases bioethanol production costs. Globally, the VHG process appears to be feasible although in need of further development efforts. In the present study, the potential of VHG technology in the industrial SCJ fermentation process was investigated. The industrial yeast strains *S. cerevisiae* PE-2 and CAT-1 were investigated for fermentation of up to 35 °Bx SCJ in the absence of other added carbon sources and the impacts of DAP and urea supplementation examined. The effects of the process conditions on the yeast strains, and in particular on the performance and viability, but importantly also on the accumulation of glycerol and expression of associated genes were examined and compared. The results show the high potential of VHG in industrial scale bioethanol production from SCJ and support VHG technology as a viable industrial solution for simultaneously reducing the environmental impact and improving economic gains of the sugar cane-based bioethanol industry.

## Results and discussion

### SCJ fermentation in VHG conditions

#### Industrial fermentation process simulation

The most common yeast strains used in the Brazilian bioethanol industry, *Saccharomyces cerevisiae* PE-2 and CAT-1 [10, 11] were examined in a simulated industrial fermentation process for bioethanol production in VHG conditions. The sugarcane juice (SCJ) was concentrated to 25, 30 and 35 °Bx, as compared to regular fermentation conditions which do not exceed ~ 20 °Bx. Fermentations were allowed to proceed for 6 consecutive cycles of 24 h, in which the yeast was reused from one cycle to the next. This is identical to the industrial process, except that the cells were not acid washed between each cycle [1, 2, 6, 38] as it is expected that the harsher conditions of VHG would reduce the potential for contamination and hence eliminates the necessity for this step [6, 38]. Broth sterilization ensured the absence of bacterial contamination during the laboratory fermentations. The assessment of fermentation progress was made by (i) measuring the amounts of ethanol produced and unfermented sugar remaining; (ii) quantifying the production of biomass wet weight and (iii) determining yeast viability at the end of each 24-h fermentation cycle.

The amounts of ethanol produced and sugar consumed by PE-2 and CAT-1 are shown in Table 1 and these values were used to estimate the corresponding ethanol yields and productivities shown in Table 2. At 25 °Bx, CAT-1 produced ~ 5% more total ethanol (sum of the 6 cycles) than PE-2 (Table 1), but upon tightening VHG conditions further, to 30 °Bx, both yeast strains produced ~ 5% less ethanol than at 25 °Bx, Interestingly, at 35 °Bx, the behaviour of the two strains diverged as PE-2 showed a further 5% decrease in total ethanol, whereas ethanol production by CAT-1 increased up to an aggregated value of 145 ml L^−1^ (Table 1). On the other hand, the amount of sugar consumed did not vary proportionally with ethanol production (Table 1), indicating fluctuations in yeast fermentation ability which translated into fluctuations in ethanol production yield (Table 2). The highest yield obtained for PE-2 was at 30 °Bx and for CAT-1 was at 25 °Bx, which do not coincide with the best ethanol producing conditions that were, respectively, 25 and 35 °Bx (Table 1). On the other hand, productivity, which corresponds to the rate at which the fermentations proceed, did not differ substantially from one strain/Bx combination to the other, with the highest productivity for each strain being obtained with the same conditions that presented highest yields (Table 2).

**Table 1:** Ethanol production and sugar consumption for sugar cane juice fermentations with the industrial yeast strains *S. cerevisiae* PE-2 and CAT-1 at 25, 30 and 35 °Brix VHG conditions

Cycle		PE-2						CAT-1					
		Ethanol (ml L^−1^)			Consumed sugar (g L^−1^)			Ethanol (ml L^−1^)			Consumed sugar (g L^−1^)		
°Brix		25	30	35	25	30	35	25	30	35	25	30	35
Fermentation cycle	1	98.8 cC	80.2 bE	62.2 cE	nd	nd	nd	109.0 cC	78.2 bC	132.1 abC	nd	nd	nd
	2	137.9 bB	123.4 bD	107.0 cD	250.3 cA	203.2 bA	159.43 aB	142.0 bB	120.6 cB	148.2 aA	237.5 cA	234.2 bA	253.0 aA
	3	144.3 aA	132.2 cC	117.3 cC	nd	nd	nd	152.0 aA	131.9 aA	152.1 aA	nd	nd	nd
	4	144.5 aA	141.4 bB	126.4 bB	248.6 cA	233.0 bB	194.44 aD	151.4 aA	132.7 cA	149.5 aA	245.4 cAB	253.0 bB	258.0 aA
	5	144.0 aA	149.7 bA	132.5 bA	nd	nd	nd	150.7 aAB	128.2 cA	147.7 aA	nd	nd	nd
	6	144.3 aA	149.9 bA	129.5 bAB	253.0 cA	247.7 bC	195.99 aD	151.5 A	129.4 cA	138.7 bB	247.1 cAB	250.7 bB	256.3 aA
AVERAGE	(ml L^−1^)	135.6	129.5	112.5	–	–	–	142.4	120.2	144.7	–	–	–
SUM	(ml)	8138	7768	6749	–	–	–	8543	7210	8683	–	–	–

Results are given for the end of each 24-h fermentative cycle and are the average of three independent assays. Significantly different values are identified by different letters. Different lower case letters indicate statistical differences within each fermentation batch, and different capital letters indicate statistical differences between different fermentation batches. Italics: maximum ethanol concentration produced by each strain. The aggregated cumulative value of ethanol produced at the end of the full 6 cycles is also shown (ml) as well as the corresponding relative production in  ml L^−1^

*nd* not determined

**Table 2:** Productivities and fermentative yields for sugar cane juice (SCJ) fermentations with the two industrial yeast strains *S. cerevisiae* PE-2 and CAT-1 at various °Brix values

SCJ °Brix	Yield (%)		Productivity (g L^−1^ h^−1^)	
	PE-2	CAT-1	PE-2	CAT-1
25	88.09 ± 0.06	94.62 ± 0.14	4.26 ± 0.17	4.98 ± 0.12
30	93.46 ± 0.22	79.76 ± 0.43	4.93 ± 0.11	4.26 ± 0.06
35	85.57 ± 0.80	83.54 ± 0.71	4.26 ± 0.10	4.56 ± 0.18

Results were calculated as described in the Methods section using values obtained at the end of the sixth 24-h cycle. Results given are the average plus or minus the standard deviation of three independent assays

**Fig. 1:**
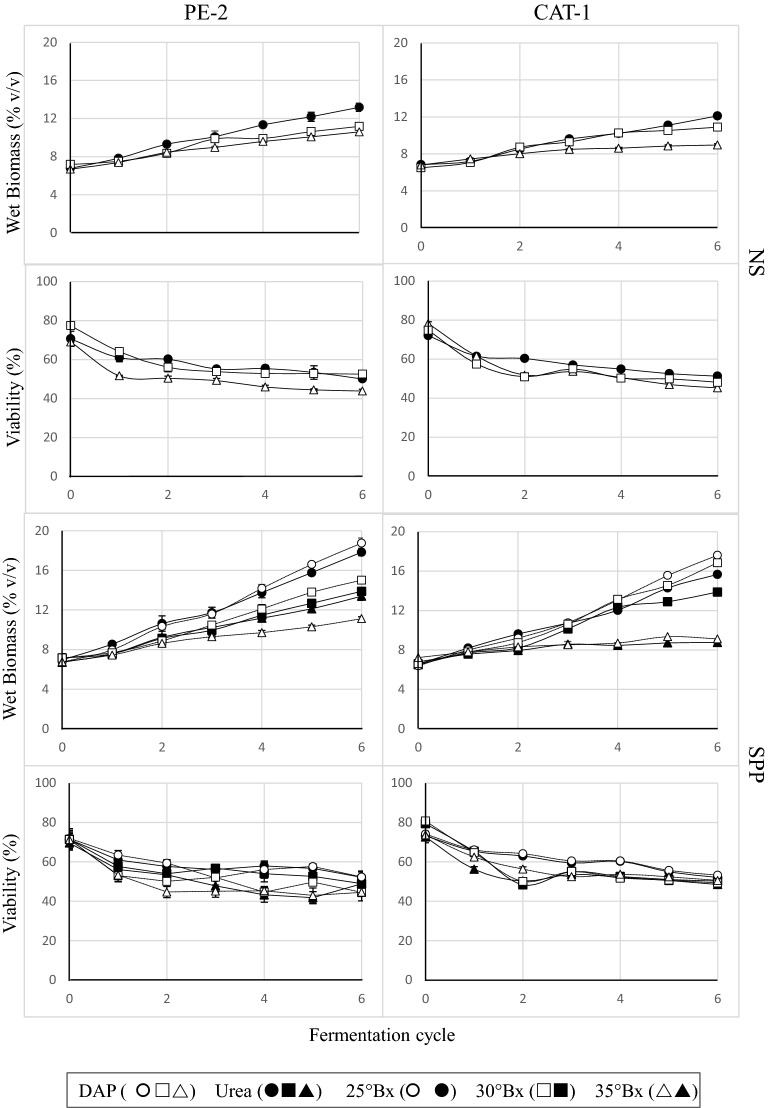
Biomass production (wet weight) and cell viability (%) for SCJ-VHG fermentations with industrial yeast strains *S. cerevisiae* PE-2 and CAT-1. Parameters were monitored during six consecutive fermentation cycles. SCJ-VHG conditions of 25, 30 and 35 °Bx without (NS), and with (SPP), DAP or urea supplementation were used. Results presented are the PP with standard deviation of three independent productions

Biomass production, as expected, increased steadily and cumulatively from cycle to cycle in all strain/Bx conditions examined (Fig. 1, upper panel, NS; NS stands for non-supplemented SCJ). At the same time, viability decreased to ~ 50% during the first 2–3 cycles before subsequently decreasing much more slowly (Fig. 1, lower panel, NS). Noticeably, inocula prepared as in industry from commercially available dry yeast, already contained approximately 20% non-viable cells. Furthermore, it can also be seen from Fig. 1 (upper panel, NS) that biomass accumulation is differently affected by the VHG fermentation conditions used. At the end of the 6 cycles at 30 and 35 °Bx, the PE-2 strain presented, respectively, 15 and 20% less biomass than with the 25 °Bx condition. For strain CAT-1, however, it can be seen that wet biomass concentrations decreased only 10% at 30 °Bx but decreased as much as 26% at 35 °Bx as compared to the standard (25 °Bx) condition.

An important indicator of process suitability for industrial application is the amount of residual sugar remaining after fermentation is terminated in a feasible period of time. The non-fermented sugar should ideally tend to zero. This was quantified for all strain/Bx combinations tested, and indicated that the remaining sugar at the end of the sixth cycle increased linearly with the Brix degree of the broth (PE-2 corr. 0.9964; CAT-1 corr. 0.9999). The rate of increase, 10.3 ± 1.5 g L^−1^°Bx^−1^, was identical for the two strains, and the maximum amount of non-fermented sugar reached ~ 40% with PE-2 at 35 °Bx. Such an increase in non-fermented sugar concentration shows the negative impact of the harsh VHG conditions on the yeast performance. Interestingly, in opposition, the amounts of sugar that were consumed by each strain at the three different °Bx (Table 1) were very similar (the global average and standard deviation of results from sugar consumed in Table 1 is 241 ± 17 g L^−1^). This apparent *consumption ceiling* could be due to the maximum amount of ethanol that the yeasts can endure while still remaining metabolically active. In the present study, ethanol production reached a maximum of ~ 15% (v/v) (Table 1, italics); ~ 14% (v/v) of which was already reached in the first cycle of fermentation and which was accompanied by a strong reduction in cell viability (Fig. 1). This viability-impacting ethanol threshold has in fact been previously reported for PE-2 [39]. Interestingly, even though this ethanol concentration was surpassed during the subsequent cycles, cell viability did not continue decreasing as steeply (Fig. 1). Indeed, although strains PE-2 and CAT-1 are reported as being capable of resisting up to 15% (v/v) ethanol under laboratory conditions [10, 39], in regular industrial fermentation conditions these strains do not produce more than 7–11% (v/v)/cycle [40].

The bioethanol industry employs 8 h cycles which go on uninterruptedly for several months, with the yeast being reused from one cycle to the next [1, 2]. This recycling of the yeast provides a means for confined evolution and selection, favouring the acquirement of resistance to the harsh specific fermentative conditions [10, 11]. In the industry, the initial stages of a sugarcane harvest season usually encompass the most variable stages of the process [40]. Theoretically, this could derive from large microbial predominance changes in the fermentation microbiome. Currently, there is no consensus as to whether the inoculated industrial strains such as PE-2 and CAT-1 do fully dominate the inoculum throughout recycling [1, 10], justifying stable and repetitive fermentation results, or if, in opposition, they are replaced by other endogenous strains present at each biofuel plant [41]. The present results show that without yeast strain variation, the first two–three cycles are the most variable (Table 1), consistent with a more severe loss of viability by the yeast cells during these cycles (Fig. 1).

### SCJ-VHG fermentation supplementation with a nitrogen-rich source

The supplementation of VHG fermentations with a nitrogen-rich nutrient supply has been investigated before [7, 8, 42]. Casamino acids, ammonium sulphate, peptone, yeast extract and urea were tentatively used in several fermentative conditions, with several yeast strains, including PE-2, with varying success. Nevertheless, all these studies were performed with synthetic laboratory media/conditions, with monitoring of a single fermentation cycle, and thereby cannot be easily extrapolated to the reality of the industrial process. Therefore, in the present study, the industry mimicking SCJ fermentation process was carried out at 25–35 °Bx with and without supplementation of the broth with a rich source of ammonium: urea (16 mM (0.045% N) as in Jones and Ingledew [7]), or 24 mM (0.067% N) di-ammonium phosphate (DAP). Although this latter is higher than the reported threshold for triggering NCR [26, 29, 34], it is recognized that this is highly variable from strain to strain [26, 43].

The sugar consumed and ethanol produced (Table 3), and the yeast biomass produced and its viability (Fig. 1) were monitored during six fermentation cycles as above (Table 1, Fig. 1). Comparing with the non-supplemented fermentations (Fig. 1, lower panel, NS), cell viability did not change significantly upon nitrogen supplementation (Fig. 1, lower panel, SPP). On the other hand, biomass production increased significantly (Fig. 1, upper panel, SPP), in particular with DAP supplementation. At 25 and 30 °Bx, CAT-1 produced 45 and 55%, and PE-2 produced 42 and 34% more biomass than in non-supplemented SCJ. In contrast, at 35 °Bx, the biomass production remained at approximately the levels of the non-supplemented broth. The biomass increases observed could indicate a positive impact of supplementation but could also correspond to a metabolic shift towards a respiratory metabolism to the detriment of fermentation. In this case, an undesired decrease in ethanol production would have been observed. However, in this study, ethanol concentrations were maintained, and even increased in some cases. Indeed, the maximum ethanol concentrations for a single cycle were observed with 30 °Bx SCJ, supplemented with DAP in the case of PE-2 (15.8% (v/v)) and supplemented with urea in the case of CAT-1 (17.4% (v/v)) (Table 3). In these assays, CAT-1 in particular consumed more sugar (261 ± 22 g L^−1^) (Table 3), overcoming the above-mentioned ~ 250 g L^−1^ threshold of the non-supplemented process. Surprisingly, at 35 °Bx/DAP, CAT-1 was still very proficient, producing as much ethanol (~ 15% (v/v)) as PE-2 at its maximum at 30 °Bx with DAP or urea supplementation. The two strains diverge in the amounts of sugar utilised. PE-2 left 60% more unfermented sugar in urea [13.7 g L^−1^ °Bx^−1^ (corr. 0.9920)] than in DAP-supplemented SCJ [8.5 g L^−1^ °Bx^−1^ (corr. 0.9726)]. CAT-1, in both supplemented and non-supplemented conditions, left identical amounts of unfermented sugar as PE-2 in non-supplemented fermentations [10 g L^−1^ °Bx^−1^ (correlations varying from 0.91586 to 0.99997)].

**Table 3:** Effects of nitrogen source supplementation on ethanol production and sugar consumption

		PE-2						CAT-1					
		Ethanol (ml L^−1^)			Consumed sugar (g L^−1^)			Ethanol (ml L^−1^)			Consumed sugar (g L^−1^)		
°Brix		25	30	35	25	30	35	25	30	35	25	30	35
Ammonium phosphate (DAP)													
Fermentation cycle	1	106.9 aB	95.5 bE	84.9 cG	nd	nd	nd	120.8 bD	128.7 aG	130.9 aD	nd	nd	nd
	2	139.2 bA	146.7 aBC	140.6 bAB	255.1 cA	240.0 bDE	278.0 aE	148.2 aAB	153.2 aDE	151.3 aA	244.1 bB	309.0 bF	259.9 aA
	3	139.4 aA	138.0 aD	138.4 aBC	nd	nd	nd	135.3 bC	129.6 bG	150.3 aA	nd	nd	nd
	4	142.9 aA	144.4 aC	144.6 aAB	255.0 cA	243.9 bCD	291.6 aF	146.8 aAB	145.2 aEF	146.0 aAB	242.8 bAB	307.6 bEF	258.8 aA
	5	138.9 cA	157.8 aA	146.8 bA	nd	nd	nd	142.6 bABC	149.3 aEF	149.6 aA	nd	nd	nd
	6	141.6 bA	158.4 aA	142.0 bAB	255.1 cA	269.9 bF	278.8 aE	140.7 aBC	143.9 aF	145.1 aABC	237.7 bAB	299.6 bDEF	254.5 aA
AVERAGE	(ml L^−1^)	134.8	140.1	132.9	–	–	–	139.1	141.7	145.5	–	–	–
SUM	(ml)	8089	8408	7973	–	–	–	8344	8499	8732	–	–	–
Urea													
Fermentation cycle	1	112.7 aB	97.2 bE	69.6 cH	nd	nd	nd	135.6 aC	126.8 bG	136.6 aCD	nd	nd	nd
	2	142.9 aA	146.3 aBC	116.4 bF	255.1 cA	65.2 bC	184.8 aA	151.1 bA	174.4 aA	149.1 bA	244.1 cB	294.6 bDE	250.1 aA
	3	142.2 aA	142.9 aCD	125.2 bF	nd	nd	nd	149.2 aAB	79.3 bH	148.1 aA	nd	nd	nd
	4	141.0 bA	152.2 aAB	129.6 cDE	255.0 cA	65.6 bC	207.0 aC	148.8 bAB	160.9 aCD	148.8 bA	243.8 cAB	276.8 bC	256.6 aA
	5	141.2 bA	153.2 aA	132.3 cCD	nd	nd	nd	149.6 bA	165.3 aBC	147.2 bAB	nd	nd	nd
	6	139.4 bA	158.2 aA	127.7 cDE	255.2 cA	48.7 bE	211.7 aC	143.5 bABC	171.0 aAB	138.9 bBCD	244.1 cB	289.2 bCD	258.7 aA
AVERAGE	(ml L^−1^)	137.6	141.7	116.8	–	–	–	146.3	146.3	144.8	–	–	–
SUM	(ml)	8194	8500	7008	–	–	–	8778	8777	8687	–	–	–

Results are shown for ammonium phosphate and urea supplemented very high gravity sugar cane juice fermentations with *S. cerevisiae* strains PE-2 and CAT-1 at 25, 30 and 35 °Brix. The results given are for the end of each 24-h fermentative cycle and are the average of three independent assays. Significantly different values are identified by different letters; different lower case letters indicate statistical differences within each fermentation batch, whereas different capital letters indicate statistical differences between different fermentation batches. Italics: maximum ethanol concentration produced by each strain. The aggregated cumulative value of ethanol produced at the end of the full six cycles is also shown (ml) as well as the corresponding relative production in   ml L^−1^

The productivities and yields of the fermentations were also found to vary with the yeast strain, °Bx and supplement used (Table 4), with the most striking result being the increase in productivity obtained for CAT-1 at 30 °Bx upon urea supplementation. Furthermore, urea addition increased the yield with CAT-1 by 14% [from 79.76% (Table 2) to 91.32% (Table 4)], while DAP caused the opposite effect, decreasing 16% [from 79.76% (Table 2) to 66.73% (Table 4)]. High yields and productivity were thus observed for CAT-1 with 30 °Bx/urea, which matched the highest ethanol production of 17.44% (v/v), leaving only 0.2% (v/v) non-fermented sugar. Such results point to the suitability of this VHG process under these conditions for industrial application. Interestingly, in non-supplemented 25 °Bx SCJ fermentations, CAT-1 was in fact advantageously able to consume all sugar present, but the amount of ethanol achieved did not increase above 15% (v/v). In opposition, PE-2 produced the highest ethanol concentrations (Table 3) and highest productivity (Table 4) at 30 °Bx with DAP, with the yield being slightly higher at the same °Bx in the absence of nitrogen supplementation (Table 2). At each of these conditions, PE-2 left 8% and 5% of the sugar unfermented.

**Table 4:** Comparison of productivities and fermentative yields for sugar cane juice (SCJ) fermentations with the two industrial yeast strains *S. cerevisiae* PE-2 and CAT-1 at various °Brix values following supplementation with ammonium phosphate or urea

SCJ °Brix	Yield (%)		Productivity (g L^−1^ h^−1^)	
	PE-2	CAT-1	PE-2	CAT-1
Ammonium phosphate				
25	85.67 ± 0.02	91.35 ± 0.03	4.65 ± 0.15	4.63 ± 0.30
30	90.63 ± 0.33	66.73 ± 0.15	5.20 ± 0.26	4.26 ± 0.21
35	78.67 ± 0.77	88.03 ± 1.74	4.67 ± 0.11	4.77 ± 0.15
Urea				
25	84.42 ± 0.05	90.81 ± 0.06	4.58 ± 0.02	4.72 ± 0.47
30	90.25 ± 0.42	91.32 ± 0.43	4.92 ± 0.11	5.63 ± 0.28
35	93.17 ± 5.18	83.26 ± 5.96	4.19 ± 0.19	4.57 ± 0.02

Values were calculated as described in the Methods section using results obtained at the end of the sixth 24-h cycle. Results given are the average plus or minus the standard deviation of three independent assays

Nitrogen source supplementation did not alter viability but both PE-2 and CAT-1 displayed continued growth (Fig. 1) even in VHG conditions at increasing Brix, while also maintaining high fermentation yields. Moreover, the quantity of sugar left unfermented, though increasing linearly with the °Bx, was still maintained low, even at strict fermentation conditions as high as 35 °Bx. Neither PE-2 nor CAT-1 were inhibited by these VHG conditions. In fact, at 35 °Bx/urea, PE-2 presented the extraordinary yield of 93% with a matching productivity of 4.19 g L^−1^ h^−1^ (Table 4). Still, when all is taken into consideration, the best performance in VHG conditions was for CAT-1 at 30 °Bx with SCJ supplemented with urea: (a) highest ethanol production (17% (v/v)—Table 3), (b) low residual unfermented sugar (0.2% (v/v)—not shown), (c) high ethanol yield (91.32%—Table 4), (d) highest productivity (5.63 ± 0.28 g L^−1^ h^−1^—Table 4), and (e) high yeast viability after 6 cycles of 24 h (approximately 60% of the initial fraction of the viable inoculum—Fig. 1).

### Effects of VHG conditions on yeast cell glycerol levels

VHG conditions correspond to extremely high osmotic stress. This is due to the very high concentrations of sugars and biomass in the fermentative broth, but also to the progressive accumulation of ethanol, which is a chaotropic agent. Yeasts accumulate glycerol as an osmolyte, to counteract this kind of stress [16]. No information is currently available of the amounts of glycerol produced or accumulated by *S. cerevisiae* strains PE-2 or CAT-1 during bioethanol production. Therefore, in the present study, glycerol production, secretion and retention during SCJ fermentation were investigated for the best performing condition identified, i.e. strain CAT-1 at VHG conditions of 30 °Bx supplemented with urea. As controls, strain CAT-1 in non-supplemented and DAP-supplemented 30 °Bx SCJ as well as in YP medium with 30% (w/v) sucrose (YPS) were monitored. In addition, laboratory strain W303-1A was also used as control due to the in-depth knowledge available in relation to the glycerol metabolism and transport mechanisms of this strain (reviewed by [13]). A single fermentation cycle of 24 h was monitored.

**Fig. 2:**
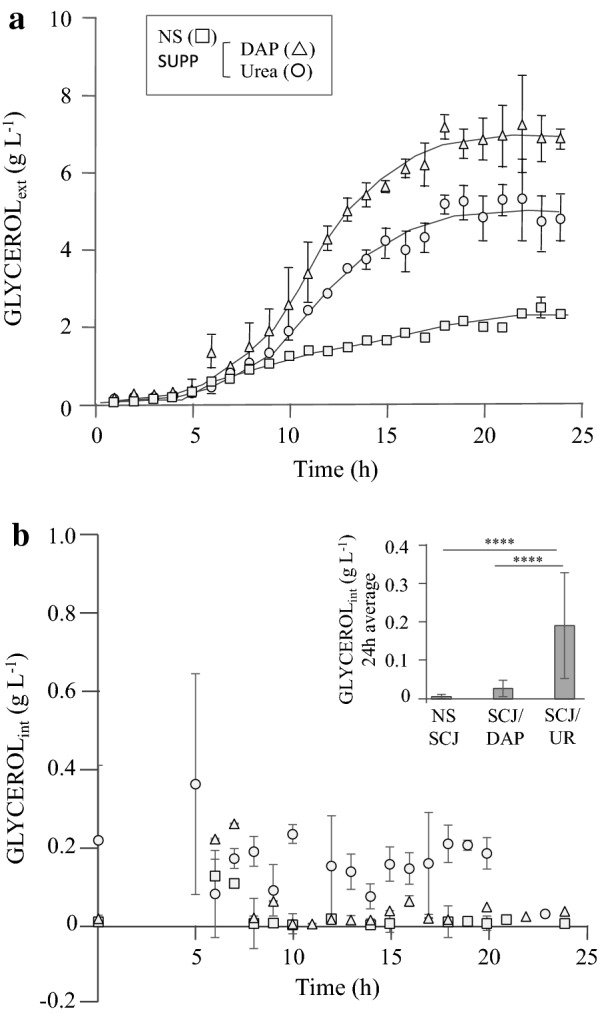
Extracellular and intracellular glycerol concentrations for SCJ-VHG fermentations with *S. cerevisiae* industrial yeast strains PE-2 and CAT-1. Glycerol concentrations were quantified during a single 24-h fermentation cycle with 30 °Bx sugar cane juice, either non-supplemented (NS, square) or supplemented (SPP) with DAP (triangle) or urea (circle). Extracellular (ext) glycerol concentrations were measured in the fermentation broth after removal of the yeast biomass (**a**), and the biomass was used for estimation of the intracellular retention (int) of glycerol (**b**). Insert: The 24-h averages of the intracellular glycerol concentrations in the non-supplemented (NS) and DAP or urea supplemented 30 °Bx SCJ fermentations are compared. Results are presented as averages with standard deviation of three independent batch cultures. ****Indicates differences with statistical significance of *P* < 0.001

Extracellular glycerol concentrations were observed to increase over time (Fig. 2a), this being more rapid during the first ~ 18 h and higher in nitrogen-supplemented broth. The biomass wet weight during the same period did not increase (not shown), confirming that the measured amounts of glycerol were derived from metabolism and not from increasing amounts of biomass. Importantly, the ammonium source promoting highest glycerol secretion to the extracellular environment was DAP, not urea (Fig. 2a). This suggests that more glycerol may be produced in DAP-SCJ and agrees with the lower ethanol concentrations quantified for this condition (Table 3). Nevertheless, as mentioned earlier, extracellular glycerol concentrations do not depend exclusively on metabolism, but crucially also on the ability of the cell to retain the glycerol intracellularly. Therefore, we also attempted to quantify the amounts of glycerol retained by the cells. Strains CAT-1 (Fig. 2b) and W303 (not shown), identically cultured on 30 °Bx SCJ or 30% (w/v) YPS, accumulated equal, albeit low, amounts of intracellular glycerol when cultivated without nitrogen supplementation and with DAP supplementation. On the other hand, when supplemented with urea, CAT-1 accumulated 4 times more glycerol than W303-1A (not shown), 7 times more glycerol than in DAP-supplemented fermentations and 28 times more glycerol than with non-supplemented fermentations (Fig. 2b). These results indicate that (1) CAT-1 produces more glycerol in VHG nitrogen-supplemented SCJ, (2) urea supplementation enables more efficient glycerol retention and (3) DAP supplementation results in higher secretion (Fig. 2). Moreover, results with YPS (not shown) did not mirror those with SCJ, suggesting that SCJ has constituents other than the sugar which are capable of influencing fermentation performance.

Retention of glycerol in the intracellular environment is believed to be achieved by the concerted action of two proteins; the Fps1 aquaglyceroporin which mediates glycerol efflux [15, 44], and the glycerol active transporter Stl1 [18] which mediates glycerol influx. Unlike other aquaglyceroporins, Fps1 controls permeation of several compounds in addition to glycerol but not urea (reviewed by [45]). Concurrently, Krenc et al. [46] showed that *S. cerevisiae* overexpressing *FPS1* grew better in nitrogen-supplemented conditions, namely with urea. This agrees with the results above (Fig. 2) in which glycerol retention was higher in urea-SCJ while glycerol secretion was higher in DAP-SCJ, and suggests that Fps1, as the biggest contributor to glycerol retention [24], could also be regulated by the nitrogen source/NCR, in addition to the HOG and CWI pathways [45, 47]. Nevertheless, while the expression of *FPS1* is primarily considered as being constitutive [48], it can be regulated [24, 47, 49], namely in response to amino acids availability and osmotic stress. Whether DAP and/or urea can identically contribute to the regulation of *FPS1*/Fps1 is presently unknown. On the other hand, the *STL1* gene, encoding a glycerol active permease, is complexly regulated [18, 50–54], most prominently responding to carbon source and stress.

**Fig. 3:**
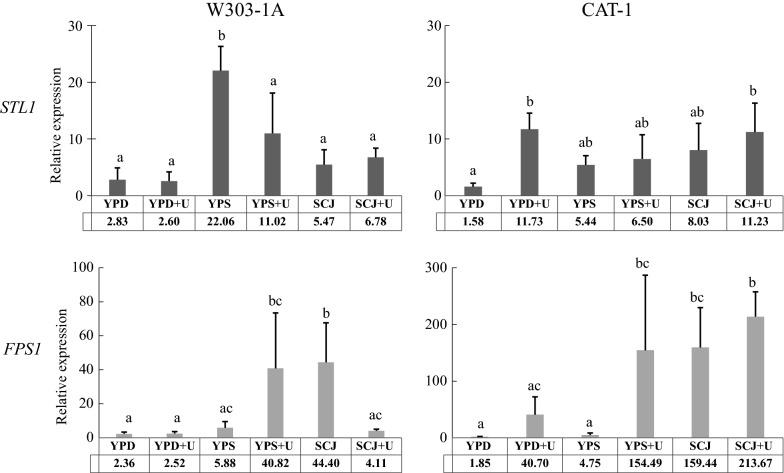
Effects of production medium composition on the relative expression of genes *STL1* and *FPS1* in *S. cerevisiae* strains CAT-1 and W303-1A. Yeast strains were grown to the mid-exponential phase in SCJ 30 °Bx and in the control media YPD and 30 °Bx YPS, and expression of the genes encoding the glycerol active permease (*STL1*) and the aquaglyceroporin (*FPS1*) quantified by qRT-PCR. The ratios of each gene expression against the expression of *ACT1* are indicated under the *x*-axis. Results are presented as the average and standard deviation of three independent batch cultures. Statistically significant differences are indicated by designation with different letters

Presently, no molecular data is available for the genes *FPS1* and *STL1* in the *S. cerevisiae* strain CAT-1. In fact, in view of the genetic diversity that this industrial strain displays [10, 11, 55], the number of alleles of *FPS1* and *STL1* in the CAT-1 genome is unknown. To assess the expression pattern of these genes, CAT-1 was cultivated as before in 30 °Bx SCJ, YPS and YPD with and without urea supplementation. The identically cultivated W303-1A strain was again used as a control. Cells were grown to the mid-exponential growth phase and the expression of both *FPS1* and *STL1* analysed by qRT-PCR. The results in Fig. 3 show the relative expression of the genes in the different conditions in relation to the lowest levels of gene expression obtained for each strain/gene combination (the base level). This strategy was previously used to analyse the expression of *STL1* and *FPS1* in the initial stages of grape must fermentations [48].

The qRT-PCR results in Fig. 3 show the compared mRNA expression of *STL1* and *FPS1* by CAT-1 in several conditions using the lowest expression value of all results as comparison standard, i.e. *FPS1* expression by YPD-grown CAT-1. Globally, the expression of *STL1* in both strains varies less than that of *FPS1*, in opposition to studies using wine strains and fermentation conditions [24, 48]. Additionally, *STL1* expression in CAT-1 was found to be higher than of *FPS1* under all growth conditions investigated (not shown). On the other hand, W303-1A grown on SCJ showed higher expression of *FPS1* than of *STL1*, which was remarkably reversed upon supplementation with urea (Fig. 3), while growth on YPS showed the completely opposite results. Interestingly, Fig. 3 also shows that growing the cell on YPS does not, at all times, mimic the growth on SCJ, corroborating that mentioned earlier that SCJ, in addition to sucrose, contains components that can influence not only the production of glycerol but also expression of *FPS1* and/or *STL1*.

Previous studies have shown that the synthesis of *STL1* mRNA was boosted upon glucose exhaustion in response to the transcription factor Cat8 of the carbon catabolite response [54]. Nonetheless, these authors also showed that *STL1* mRNA was still present, albeit at low concentrations, in the presence of glucose and in the absence of Cat8. Concurrently, an equally low but not absent expression was also detected by Noti et al. [48] in winemaking yeast strains exponentially growing on YPD, i.e. under glucose repression [18]. The present results (Fig. 3) agree with this in that *STL1* does not appear to be under such a tight glucose repression as previously described. One possibility is that the very low *a*_w_ stress conditions of the VHG fermentations might overcome glucose repression of the *STL1* promoter, as previously observed with high temperatures [56]. In addition, the high amounts of ethanol and glycerol accumulated during the fermentation cycle could contribute to alleviation of the repression exerted by the sugars. Finally, it can be seen that the very low *STL1* expression in YPD compares well with the low expression observed in the work of Noti et al. [48] and Haurie et al. [54]. Regardless of the relative contributions of Stl1 and Fps1 to the intracellular accumulation of glycerol as measured in the present work, it is also plausible that glucose promoted endocytic removal of Stl1 from the membrane [18] might also be alleviated.

**Fig. 4:**
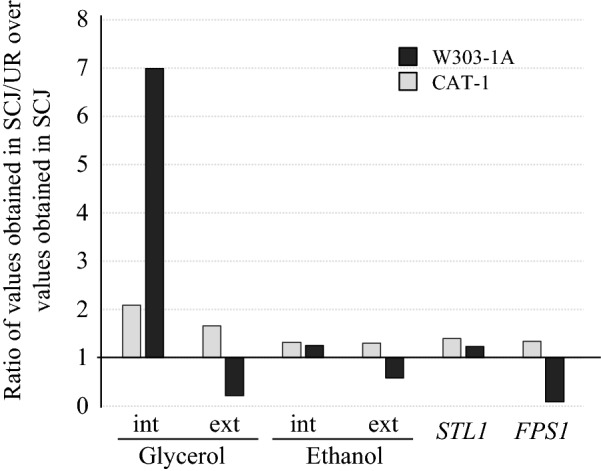
Effects of urea supplementation of 30 °Bx SCJ-VHG fermentations on glycerol and ethanol concentrations and gene expression in *S. cerevisiae* strains CAT-1 and W303-1A. Results for intracellular (int) and extracellular (ext) glycerol and ethanol concentrations and *STL1* (glycerol active permease) and *FPS1* (aquaglyceroporin) expression, by CAT-1 (light grey bars) and W303 (dark grey bars), are shown as ratios of the mean values obtained for SCJ urea-supplemented (SCJ-UR) to SCJ non-supplemented (SCJ) fermentations

Effectiveness in the bioethanol industry requires maintenance of high ethanol production and secretion throughout the process. In this regard, *FPS1* was previously found to be determinant in ethanol secretion and resistance to ethanol-induced stress [39]. Its deletion and overexpression, respectively, increased and decreased the intracellular accumulation of ethanol [39], supporting the suggestion that *FPS1* could mediate the exit of ethanol. If this is the case, it would have to be meticulously orchestrated with the retention of glycerol. Closing the channel would promote glycerol retention/osmotic stress resistance, allowing healthy active metabolism, but also leading to accumulation of ethanol. Opening it would prevent intracellular accumulation of ethanol to toxic levels [57, 58], but would also free glycerol, thereby decreasing osmotic stress resistance. Panchal and Stewart [59] showed that regardless of the relative production of ethanol and glycerol during brewing, the intracellular concentration of both compounds decreased similarly during the fermentation process, in particular following the first 24 h, though glycerol appeared to be secreted faster. This allows one to hypothesise that ethanol only leaves the cell when glycerol is secreted and suggests that the Fps1 channel might regulate the exit of either compound according to differences in affinity. The present results corroborate this interpretation by showing that when *FPS1* expression decreases significantly, as observed with W303-1A cultivated in SCJ-urea compared to SCJ alone (Fig. 3), the amounts of glycerol secreted decrease proportionally, as well as those of ethanol (Fig. 4). The decrease in excreted glycerol was higher than that observed for ethanol, as would be expected if the channel’s affinity for exporting glycerol was higher than that for exporting ethanol. On the other hand, the variations in expression of *STL1* observed in both W303-1A and CAT-1 are comparable to those for the intracellular retention of glycerol in both strains (Fig. 4), as might be expected from its role as a glycerol influx-mediating high affinity permease. The very high increase in intracellular glycerol concentration observed in W303-1A upon urea supplementation (Fig. 4) should derive from a strong stimulation of metabolism by urea but has not been reported previously. CAT-1 displayed smaller variations, though importantly the expression of both *STL1* and *FPS1* increased about 30% in the presence of urea (Fig. 4), in agreement with the relative amounts of extracellular and intracellular glycerol and ethanol.

### Final remarks

Yeast strain CAT-1 fermenting VHG 30 °Bx SCJ with urea supplementation performs better than: (i) in non-supplemented VHG conditions, (ii) strain PE-2 in all conditions tested, and (iii) either strain in less harsh VHG conditions (25 °Bx). Importantly, in these conditions, CAT-1 produced 17% (v/v) ethanol, with a residual unfermented sugar concentration of only 0.2% (w/v). Moreover, the extreme osmotic stress of VHG conditions was compensated for by production and retention of high amounts of glycerol, with this being higher upon urea supplementation. DAP supplementation in opposition promoted a higher secretion of the glycerol produced. More efficient retention of glycerol thus appears to be a requisite for enhanced yeast viability and fermentation performance. The differences in glycerol retention by strain CAT-1 in DAP or urea supplemented SCJ agrees with the expression paterns observed for the two genes encoding the glycerol permease Stl1 and channel Fps1 in these conditions. The results also show that urea may promote the deregulation of *STL1* expression and overcome glucose repression. Moreover, the secretion of glycerol by Fps1 increases moderately with increased expression of the *FPS1* gene. This was also observed with ethanol, corroborating previous suggestions that Fps1 might play some role in ethanol exit from the cell [39].

This work clearly shows that the application of VHG technology to SCJ industrial fermentations is feasible, provided that the fermentation broth is supplemented with urea and that an industrial, robust yeast strain such as CAT-1 is used. The fermentation yield and productivity and associated yeast viability observed in this study are at levels suitable for industrial application and the use of these VHG conditions should significantly reduce the environmental impact and cost of the SCJ bioethanol production process.

## Methods

### Fermentations

#### Microorganisms

*Saccharomyces cerevisiae* industrial strains CAT-1 and PE-2 [11] were purchased from LNF Latino Americana, Bento Gonçalves/RS, Brazil (http://www.lnf.com.br/index.html). The lyophilised-dried yeast was rehydrated with H_2_O at 30 °C (3 g 20 mL^−1^) with mild shaking at 70 rpm for 15 min. The suspension was subsequently centrifuged (Damon/IEC BP-6000) at 2250 rpm for 10 min. and the supernatant discarded with the cells being used as inoculum. The *S. cerevisiae* laboratory strain W303-1A MATa/MATα {leu2-3, 112 trp1-1 can1-100 ura3-1 ade2-1 his3-11, 15} [phi+] was also used. This strain was maintained in YPD 2% agar at 4 °C, and cultured in YPD batch cultures at 30 °C with agitation at 70 rpm (New Brunswick Scientific Co., US) to obtain biomass for SCJ inoculation.

#### Must preparation

Fermentation must was prepared from sugarcane variety SP83-2487 (Department of Agro-Industry and Nutrition, ESALQ, Piracicaba/SP, Brazil). The cane was grinded, passively filtered through cotton to withdraw the solid particles, and concentrated up to a maximum of 37% (w/v) by vapour heating. The industrial designation of *Brix degree* (Brix; °Bx) is used to designate this percentage, i.e. the number of g of soluble sugar present in 100 g of SCJ. Brix degree was checked by refractometry (Megabrix RFBZW25). Following concentration, the sugarcane juice was cooled to room temperature, filtered in the same manner as above to remove the solid residues, and sterilised by autoclaving at 121 °C, 1 atm for 30 min. SCJ was stored at 10 °C. Prior to each fermentation assay, the concentrated juice was diluted in distilled water to the desired concentration, i.e. 25, 30 or 35 °Bx.

#### Industrial fermentation process simulation

A reduced scale fermentation procedure which mimics the industrial fermentation process used in the Brazilian bioethanol industry was utilised. Yeast wet biomass (7 g) was gradually supplemented with must, prepared as described above, at 20 mL h^−1^ for 5 h. Both non-supplemented and 16 mM urea ((NH_2_)_2_CO) or 24 mM diammonium phosphate (DAP) ((NH_4_)_2_HPO) supplemented musts were used. Fermentations were carried out with ~ 10^8^ cells (enumerated with a Neubauer chamber and Leica light microscope) in 100 mL broth, in 150 mL tubes, for 24 h at 30 °C with orbital agitation at 70 rpm (New Brunswick Scientific Co., US), followed by centrifugation (Damon IEC BP-6000) at 2250 rpm for 10 min. The cell pellet was weighed and supplemented with an identical number of grams of fresh must, followed by a new 24-h fermentation cycle, and this whole process was repeated 6 times. The supernatant of each cycle was stored at − 20 °C for further analysis. Assays were performed in triplicate. Additionally, as controls, identical batch cultures were performed in YP medium with 30% (w/v) sucrose (YPS).

#### Assessment of yeast cell viability

Yeast cells viability at the end of each 24-h fermentation cycle, prior to centrifugation, was determined with the erythrosine staining method [60]. Viable unstained cells and non-viable red cells were counted in a Neubauer chamber with an optical microscope (Nikon Alphashot). Results are presented as the ratio of the two cell numbers.

*Compositional analysis* (1) Ethanol concentration in the fermentation broth was determined by densitometry (Anton Paar DMA 4500) after distillation by steam drag (Tecnal TE-012). (2) Concentrations of sucrose, glucose and fructose were determined by high-performance liquid chromatography (ICS 2500, HPLC Dionex) with amperometric detection (ED50) equipped with an autosampler AS50. Sugars were assigned according to the retention times of standards (sucrose, glucose and fructose). A Carbopac PA-1 column (4 × 250 mm, Dionex) and a guard Carbopac PA-10 column (4 × 50 mm, Dionex) were used. The mobile phase was 100 mM NaOH at a flow rate of 0.9 mL min^−1^. All samples were treated with 2% (v/v) perchloric acid and filtered through a 0.22 µm membrane (Millipore) before analysis. (3) Glycerol was quantified by HPLC (VWR-Hitachi LaChrom Elite) on a Phenomenex Rezex ROA-organic acid H^+^ (8%) column at 60 °C, using sulphuric acid (2.5 mM) at a flow rate of 0.5 mL min^−1^ as the mobile phase [61]. Samples of 10 mL culture were centrifuged (7000 rpm for 2 min at 4 °C), and secreted and intracellular glycerol were quantified as previously described, using the supernatant and the cells in the pellet, respectively [62]. All samples were first deproteinised by treatment with 10% trichloroacetic acid, followed by centrifugation for 15 min at 14,000 rpm, and filtration through a 0.22-μm filter before HPLC analysis.

#### Determining fermentation yield and productivity

Ethanol yield was calculated according to the theoretical consideration that the conversion of 100 g glucose should give rise to 51.1 g, or 64.75 mL, ethanol [63]:

$${\text{Theoretical quantity of ethanol expected}}\, = \,{\text{Total fermentable sugar}}\, \times \,0. 6 4 7 5,$$

from which

$${\text{Fermentation efficiency}} = \frac{{{\text{Quantity of ethanol produced }}\left( {\text{mL}} \right)}}{{{\text{Theoretical quantity of ethanol expected }}\left( {\text{mL}} \right)}} \times 100$$

and

$${\text{Productivity}}\, = \,{\text{Fermentation efficiency }}\left( \% \right)/{\text{cycle extension }}\left( {\text{h}} \right).$$

### Quantitative real-time PCR (qRT-PCR)

#### Yeast RNA isolation

*Saccharomyces cerevisiae* CAT-1 and W303-1A strains were grown in YPD, YPS and 30 °Bx SCJ with and without 16 mM urea supplementation. Samples of ~ 5 × 10^7^ yeast cells were collected at OD_600_ ~ 1.0, and the cells mechanically disrupted with 0.5 mm diameter glass beads in a swing mill at 30 Hz for 15 min. Total RNA was extracted and isolated using the NucleoSpin^®^ RNA kit (Macherey–Nagel) and subsequently quantified using a ND-1000 UV–visible light spectrophotometer (NanoDrop Technologies). RNA quality was evaluated by agarose gel electrophoresis.

#### Quantification of glycerol permease and channel expression by qRT-PCR

Primers for qRT-PCR (Table 5) were designed using Primer3Plus software, aligned against the *S. cerevisiae* genome sequence (http://www.yeastgenome.org/blast-sgd) to confirm specificity, and analysed with the Mfold server (http://unafold.rna.albany.edu/?q=mfold) to confirm the absence of possible formation of self-folding secondary structures. Total RNA (500 μg) was reverse transcribed into cDNA using the iScript cDNA synthesis kit (Bio-Rad) and cDNA levels analysed using a Bio-Rad^®^ CFX96 Touch™ real-time PCR instrument. Each sample was tested in duplicate in a 96-well plate (Bio-Rad, CA). The reaction mix (10 μL final volume) consisted of 5 μL SsoAdvanced™ SYBR^®^ Green Supermix (Bio-Rad), 0.25 μL of each primer (250 nM final concentration) and 2 μL of the cDNA preparation. A blank control (without template) was included in each assay. The thermocycling programme consisted of one hold at 95 °C for 30 s, followed by 40 cycles of 10 s at 95 °C and 30 s at 60 °C. After completion of these cycles, a melting-curve analysis was performed (65–95 °C; 0.5 °C increments, 5 s) and data were collected to verify PCR specificity and the absence of contamination and primer dimers. Two different extracts of total RNA were analysed for each condition, with at least duplicate PCRs. The data were normalised to actin gene expression. The comparative Ct method (2^−ΔΔCt^ method) [64] was used to analyse results and the results presented for each condition tested are the mean of the two different RNA extractions.

**Table 5:** Sequence of the primers used for qRT-PCR

Primer	Sequence
STL1 Fw	5′ TCTGCGGTGAAAGAATTGG 3′
STL1 Rv	5′ TGATTGCCAAACGGGAATA 3′
FPS1 Fw	5′ ATTGATCGGTGCCTTCACA 3′
FPS1 Rv	5′ CGCAAATGTTCCTGCTTGT 3′
Actin Fw	5′ AGCCCCAGAAGCTTTGTTC 3′
Actin Rv	5′ ACCACCGGACATAACGATG 3′

#### Statistical analysis

All assays were repeated at least three times using independent culture inocula. Results were subjected to statistical variance analysis (ANOVA) and randomised block design Tukey tests with three replicates per block [65], using the Statistica v.12 programme (http://software.dell.com/br-pt) for all experiments except the qRT-PCR in which case Prism 6 (GraphPad Software, Inc.) was used. For each qRT-PCR analysis, the condition with the lowest level of gene expression was considered as the base value. Results are presented as averages with standard deviations, with significantly different values being identified by the use of different letters: different lower case letters indicate statistical differences within each fermentation batch, and different capital letters indicate statistical differences between different fermentation batches.
